# Effect of Injection Molding Melt Temperatures on PLGA Craniofacial Plate Properties during* In Vitro* Degradation

**DOI:** 10.1155/2017/1256537

**Published:** 2017-09-06

**Authors:** Liliane Pimenta de Melo, Gean Vitor Salmoria, Eduardo Alberto Fancello, Carlos Rodrigo de Mello Roesler

**Affiliations:** ^1^LEBm Biomechanics Engineering Laboratory, University Hospital (HU), Federal University of Santa Catarina, 88040-900 Florianópolis, SC, Brazil; ^2^NIMMA Laboratory of Innovation on Additive Manufacturing and Molding, Federal University of Santa Catarina, 88040-900 Florianópolis, SC, Brazil; ^3^GRANTE, Department of Mechanical Engineering, Federal University of Santa Catarina, 88040-900 Florianópolis, SC, Brazil

## Abstract

The purpose of this article is to present mechanical and physicochemical properties during* in vitro* degradation of PLGA material as craniofacial plates based on different values of injection molded temperatures. Injection molded plates were submitted to* in vitro* degradation in a thermostat bath at 37 ± 1°C by 16 weeks. The material was removed after 15, 30, 60, and 120 days; then bending stiffness, crystallinity, molecular weights, and viscoelasticity were studied. A significant decrease of molecular weight and mechanical properties over time and a difference in FT-IR after 60 days showed faster degradation of the material in the geometry studied. DSC analysis confirmed that the crystallization occurred, especially in higher melt temperature condition. DMA analysis suggests a greater contribution of the viscous component of higher temperature than lower temperature in thermomechanical behavior. The results suggest that physical-mechanical properties of PLGA plates among degradation differ per injection molding temperatures.

## 1. Introduction

Poly(lactic-co-glycolic acid), PLGA, is a biocompatible, biodegradable, and FDA-approved polymer. In the last two decades, PLGA has been considered as one of the most promising polymers for biomedical engineering applications, such as plates and screws in craniofacial surgery [1–4]. One of the techniques used in the manufacturing of medical devices is injection molding, which allows the development of complex mold geometries. However, mechanical properties stability and physicochemical properties of the resorbable material can also be strongly influenced by manufacturing process and design of the devices [5–8].

PLGA plates and screws must have suitable strength and ductility for biomechanical function, biocompatibility, and degradation. In particular, melt processing temperatures during injection molding could develop different microstructures of the manufactured device, including other operative parameters, such as mold temperature, injection flow rate, and holding pressure [9]. As semicrystalline, PLGA devices are heterogeneous systems comprised of highly anisotropic crystallites, a phase in which the chains show long-range 3D order. The size and distribution of these crystals and the viscoelasticity are extremely dependent on the molecular weight distribution and the conditions under which the material is processed [10]. In manufacturing, the parameters can affect viscosity and chain orientation during the process of molded devices, where crystallinity is an important parameter because it can increase flexural stiffness and decrease the impact properties of the final product [10, 11].

Once properties of resorbable polymers devices are established, degradation rate must be evaluated. Many factors could influence degradation rate, such as implant site, mechanical stress, molar mass distribution, chemical/stereoisometric composition, crystallinity, morphology, size and geometry of the carrier, and surface roughness [10, 12–17]. Biodegradation and reabsorption process of poly(*α*-hydroxy acids) is a succession of events. Material is initially hydrated exposed to the body's aqueous fluids. With water molecules presence, the degradation process occurs through the hydrolysis of the ester linkages, resulting in products in the form of soluble and nontoxic oligomers (or monomers). The degradation proceeds passive hydrolytic cleavage, characterized by changes in molecular weight, glass transition temperature (*T*_*g*_), moisture content, and mechanical properties, such as tensile and compressive strength [13, 18, 19]. Therefore, time for osteosynthesis must be less than the time of the mechanical properties retention.

In this work, PLGA plates were designed and manufactured by injection molding as craniofacial bioresorbable medical devices. Two different melt temperatures were tested for the injection molding process (i.e., 240 and 280°C). The high melt temperature condition was defined as the upper work limit temperature of injection molding manufacturing, while lower temperature was defined by minimum processability temperature. For both conditions, PLGA craniofacial plates were evaluated by mechanical properties (bending stiffness, flexural maximum strength, and storage modulus), physicochemical properties (crystallinity and transitions temperatures), and morphology during* in vitro* degradation.

## 2. Materials and Methods

### 2.1. Material

Poly(L-lactic-co-glycolic acid) 85/15 granules (PURASORB PLG 8531) were purchased by PURAC Biomaterials (Netherlands). The PLGA 85/15 showed an average molecular weight, Mn = 224.27 g/mol, and a polydispersity index of 1.87 (Gel Permeation Chromatography, Viscotek VE 2001, Viscotek Detector TDA 302, USA, 2008). The transition temperatures declared by the manufacturer are Tg = 57 ± 1°C and Tm = 140°C and intrinsic viscosity was 3.04 dl/g (chloroform, 25°C, *c* = 0.1 g/dl) (PURAC, 2012).

### 2.2. Plates Design and Processing

The implant was designed by 3D CAD SolidWorks 2014 software (Concord, MA). The design of the craniofacial plate device is characterized by 2 mm of thickness, 5.8 mm of width, and 38.7 mm of length and by 8 aligned holes of 2 mm semicircle screw thread. The pellets of PLGA were processed using an injection molding machine (ARBURG 270S, 250-70 model). To investigate processing influence on PLGA plates properties, two different melt injection temperatures were considered: low temperature (*T* = 240°C, PLGA_low*T*) and high temperature (*T* = 280°C, PLGA_high*T*). The other processing parameters were kept constant, as summarized in Table 1.

### 2.3. *In Vitro* Hydrolytic Degradation

Injection molded plates (0.25 ± 0.01 g per sample/plate) were desinfected by immersion in 70% v/v ethanol. Samples were immersed (*n* = 16, per each condition, per time point) in the phosphate-buffered saline (PBS) solution (0.25 g/30 ml, pH = 7.4) by storing them in the thermostatic bath (37 ± 1°C) for 15, 30, 60, and 120 days. At each time point, the degradation solution pH (pH = 7.4) was recorded and the samples were removed from the buffer solution, washed, and held in distilled water for 1 hour to remove as much buffer solution as possible. They were weighed in the wet condition and then dried in a vacuum oven at 23°C for 8 h. The samples were kept under vacuum prior to the characterization tests.

### 2.4. Dynamic Mechanical Analyses

PLGA_low*T* and PLGA_high*T* specimens were submitted to the mechanical characterization, performed by a Dynamic Mechanical Analyzer (DMA Q800, TA Instruments) at the different degradation points (i.e., 0, 15, 30, 60, and 120 days). For viscoelasticity analysis, samples (*n* = 6, gauge length = 16 mm) were tested by setting 1 Hz frequency and 0.1% relative strain of area. DMA tests were performed in the temperature range of 30–80°C at a temperature rate of 3°C min^−1^. From each test, storage modulus (*E*′), loss modulus (*E*′′), and tan⁡*δ*(*E*′′/*E*′) trends in function of the temperature were obtained and the transition temperatures were determined as peak in tan⁡*δ* trends.

For three-point bending tests, samples (*n* = 5, gauge length = 10 mm) were tested in three-point flexural mode (ASTM D790) [20], kept at 37°C, using a test speed of 2 N min^−1^. The considered mechanical parameters were bending stiffness (*E*_*f*_), maximum bending deformation (*ε*_*f*_), and flexural stress (*σ*_*f*_), calculated according to the following equation:

(1) $$\begin{array}{c} \sigma_{f}=\frac{3PL}{2bd^{2}}, \end{array}$$

where *σ*_*f*_ is stress in the outer fibers at midpoint (MPa); *P* is load at a given point on the load-deflection curve (N); *L* is support span (mm); *b* is width of beam tested (mm); and *d* is depth of beam tested (mm).

### 2.5. Differential Scanning Calorimeter Analysis

The crystallinity and thermal properties (melting point, *T*_*m*_, glass transition temperature, *T*_*g*_, enthalpy of cold crystallization, Δ*H*_*c*_, and enthalpy of melting, Δ*H*_*m*_) were obtained by using a DSC (Shimadzu DSC-6000) in a nitrogen atmosphere of 19 cm^3 ^m^−1^, using aluminum oxide as standard. The applied heating rate was 10°C min^−1^, from 10 to 250°C, using an average sample size of 7 mg taken from the central region of molded specimens (*n* = 3). Degree of crystallinity (*X*_*c*_) was determined by using the following formula:

(2) $$\begin{array}{c} X_{c}=100\times \left(\;\frac{\Delta H_{m}-{\Delta H}_{cc}}{{\Delta H}_{m}^{c}}\;\right)\times \frac{1}{1-m_{f}}, \end{array}$$

where Δ*H*_*m*_ is enthalpy of fusion, Δ*H*_cc_ is enthalpy of cool crystallization, Δ*H*_*m*_^*c*^ is heat of melt of purely crystalline PLA, taken as 93 J/g [10, 19], and (1 − *m*_*f*_) is weight fraction of the polymer in the sample. Crystallization temperature (*T*_cc_) was obtained from the second heating.

### 2.6. Fourier Transform Infrared Spectroscopy

Fourier transform infrared spectroscopy (FT-IR) was performed using attenuated total reflection (ATR) mode on a Shimadzu spectrometer, model TENSOR 27. The spectra of the samples (*n* = 3) were obtained in 4000 to 600 cm^−1^ wavenumbers by 4 cm^−1^ resolution. FTIR analysis identified bioresorbable copolymer functional groups and the possible changes due to the degradation.

### 2.7. Gel Permeation Chromatography

Molar mass distribution of the PLGA copolymer was verified by a high-performance liquid chromatography (GPC) Viscotek VE 2001 coupled to the Viscotek Detector TDA 302, Houston, Texas, USA (2008). THF solvent was used as the mobile phase and the parameters included flow rate at 1000 ml/min, injection volume of 100 ul, increment volume of 0.00333 ml, and detector and column temperature of 45°C. The injected volume was always 100 *μ*L and flow velocity was 1 cm^3 ^min^−1^. PLA samples were used as standard.

### 2.8. Scanning Electron Microscopy

SEM was used to observe the surface morphology of PLGA plates during the* in vitro* degradation. At each time point (i.e., 0, 15, 30, 60, and 120 days), PBS was removed from the samples and immersed in distilled water for 2 h. After that, the samples were kept under vacuum for SEM observation. The samples (*n* = 2) were covered with a thin layer of gold/palladium using a cathodic spray (Diode Sputtering System, International Scientific Instruments) and observed at different magnifications (original ×13) with an acceleration voltage of 10 kV in a scanning electron microscope (SEM) Jeol JSM-6390L model.

### 2.9. Statistical Data Treatment

Analysis of variance (ANOVA) was performed considering a statistical significance set at 0.05; *p* value was investigated for significance of the factors among melt temperatures. All data are reported as mean ± standard deviation.

## 3. Results

### 3.1. Mechanical Tests Analyses

The flexural stiffness, flexural strength, and maximum flexural strain of PLGA_ low and PLGA_high*T* are summarized in Table 2.

**Figure 1:**
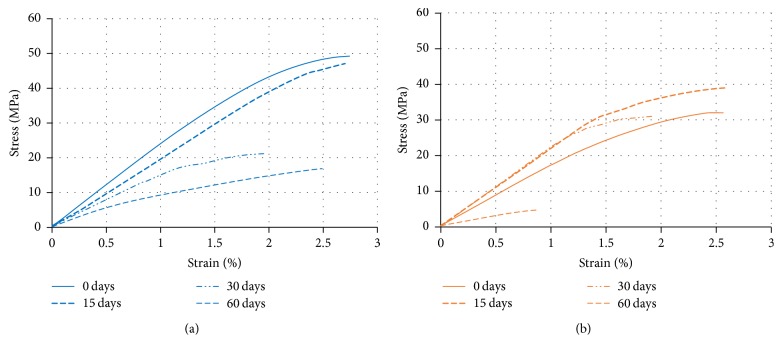
Representative stress-strain curves of PLGA_low*T* (a) and PLGA_high*T* (b) at all degradation time points obtained by flexural tests.

Flexural stiffness values,* E*, showed 2.2 ± 0.1 and 1.9 ± 0.1 GPa for plates without degradation (0 days) for PLGA_low*T* and PLGA_high*T*, respectively, and the flexural strength, *σ*_r_, was 41.4 ± 11.8 and 30.1 ± 3.1 MPa, respectively. In the flexural test, PLGA plates presented break under all conditions. This is probably due to the presence of crystallinity and chain organization during the solidification of the material in the injection molding process. PLGA_low*T* plates support additional load, exhibiting greater flexural strength at 0 days of degradation. Flexural curves for PLGA_low*T* and PLGA_high*T* during degradation are shown in Figure 1.

**Figure 2:**
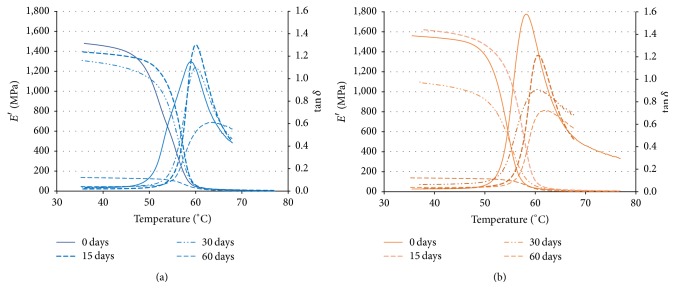
Representative curves of storage modulus (*E*′) and tan⁡*δ*(*E*′′/*E*′) as a function of the temperature obtained by DMA of the different degradation points (e.g., 0, 15, 30, 60, and 120 days) for PLGA_low*T* (a) and PLGA_high*T* (b).

Mechanical properties decrease especially after 30 days studied. DMA curves of PLGA_low*T* and PLGA_high*T* during the degradation study are shown in Figure 2. Storage modulus (*E*′) showed comparable trends and values for the two injection molding temperatures. The storage modulus *E*′ (*T*_*g*_ at 37°C) was 1.2 ± 0.2 GPa for both conditions in plates without degradation. The behavior of tan⁡*δ* was different for the two conditions processed. In particular, the highest values achieved for PLGA_high*T* are related to a higher loss modulus. This behavior suggests a greater contribution of the viscous component of PLGA_high*T* than PLGA_low*T* in thermomechanical behavior. *T*_*g*_ detected as peak in the tan⁡*δ* trend was 57.4 ± 1.8°C for both conditions.

Storage modulus *E*′ is the component related to the elastic energy stored; and *E*′′ is related to dissipated viscous energy. Both properties decreased significantly at point 3 (60 days).

### 3.2. Differential Scanning Calorimeter Analysis

Values of crystallinity (*X*_*c*_), transition temperatures (*T*_*g*_, *T*_*m*1_, *T*_*m*2_, *T*_*c*_), and enthalpies detected by DSC (Δ*H*_cc_, Δ*H*_*m*_, Δ*H*_*g*_) are shown in Table 3. The values correspond to PLGA_low*T* and PLGA_high*T*, (i.e., 0, 15, 30, 60, and 120 days) and for PLGA_pellet as a control.

The glass transition temperature occurs at 56.0 ± 0.5°C for PLGA_low*T* and at 52.7 ± 0.9°C for PLGA_high*T* at the first point studied without degradation (i.e., 0 days). The glass transition temperature of the preprocessed PLGA samples (pellet) was detected at 60.4 ± 1.8°C. Changes in transition temperatures for PLGA_low*T* and PLGA_high*T*, compared to PLGA_pellet, may be referred to changes related to the molecular chain during the injection molding processing. The endothermic melting peak of PLGA_low*T* appeared at 156.6 ± 0.2°C. Considering PLGA_high*T*, this peak occurs twice in one shoulder at 151.0 ± 2.2°C and 157.9 ± 1.1°C, due to differences in crystallinity. Percent crystallinity was measured by the calculation of (2).

The DSC curves show the difference in the endothermic peaks and the presence of high crystallinity only for PLGA_high*T*. In addition, there is shoulder presence indicating two melt temperatures (146 and 157°C), related to the PGA (15%) and PLA (85%) fractions, respectively. For PLGA_low*T*, a very mild melting endotherm occurs at 153.1 ± 0.3°C, which could indicate a second *T*_*m*_, even if this value is very close to the detected *T*_*m*_.

**Figure 3:**
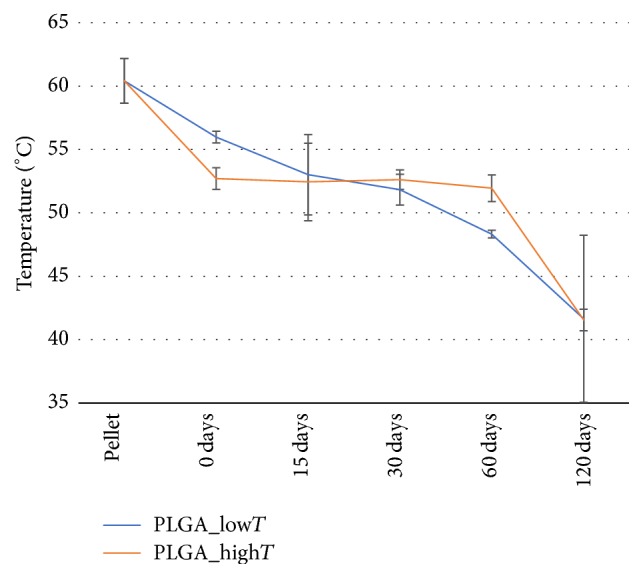
Glass transition temperatures (Tg) measured by the DSC for PLGA_low*T* and PLGA_high*T*.

The decrease presented by Tg of the copolymer (Figure 3), as a function of the degradation time, in the period from 0 to 120 days (from 56.0 ± 0.5°C with 0 days to 41.7 ± 6.6°C for PLGA_low*T* in 120 days and from 52.7 ± 0.9 to 41.6 ± 0.8°C for PLGA_high*T*) indicates rapid hydrolytic degradation of plates in PBS medium. In fact, this is characteristic of PLGA copolymers. At the beginning of the degradation process, *T*_*g*_ decrease could be associated with the plasticizing effect of the plates by the H_2_O absorption [17]. Moreover, there is an advanced degree of degradation of plates from 60 days.

### 3.3. Fourier Transform Infrared Spectroscopy

**Figure 4:**
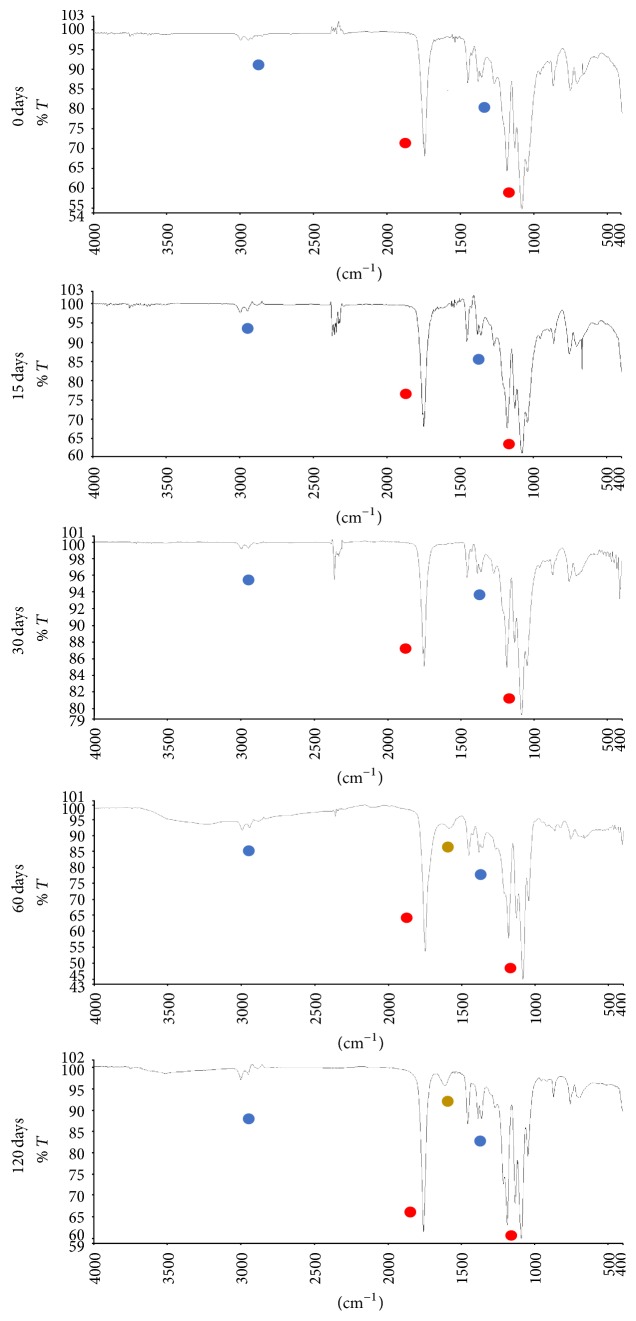
Spectrum related to degradation points 0 to 4 of PLGA plates for the period of 0, 15, 30, 60, and 120 days, respectively. Ester groups = red: 1760–1745 cm^−1^ and 1300–1150 cm^−1^; alkanes groups = blue: 3000–2800 cm^−1^ and 1450–1370 cm^−1^; oligomer groups = yellow: 1600–1500 cm^−1^.

Absorption bands were identified in the spectrum of the PLGA_low*T* and PLGA_high*T* plates at the different degradation periods: an intense band between 1760 and 1750 cm^−1^, characteristic of carbonyl (C=O), present in the two monomers, and a bonding band (C-O) between 1300 cm^−1^ and 1150 cm^−1^, characteristic of the ester groups. The absorption bands that are characteristic of the functional groups present in the PLGA copolymer can be observed in the spectra (Figure 4) and are shown in Table 4.

The presence of the O-C=O group near the absorption band 1600–1500 cm^−1^ indicates signs of degradation; note that the presence of this band occurs in 60 and 120 days of degradation in PBS solution. Usually the appearance of a band at 3400 cm^−1^ relating to vibration of OH bands in groups -COO-H and -CO-H at the ends of strings is observed, indicating a reduction in their size, and also the occurrence of the peak at 1605 cm^−1^ corresponds to the asymmetric stretch of the -COO- group in oligomers.

### 3.4. Gel Permeation Chromatography

**Figure 5:**
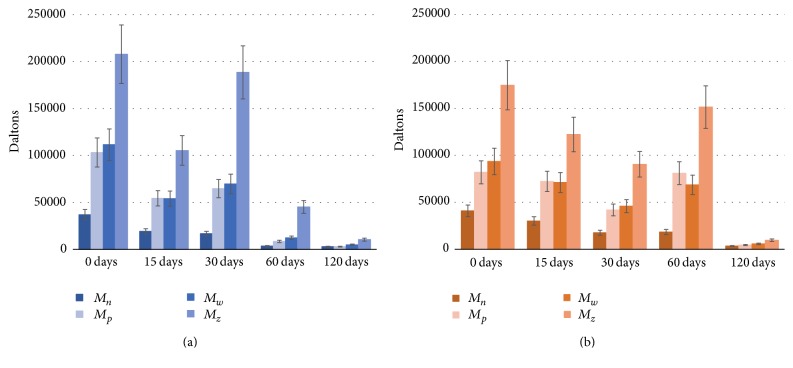
Molar mass (mean ± deviation) of the PLGA plates in the 0, 15, 30, 60, and 120 days of degradation in PBS solution for PLGA_low*T* (a) and PLGA_high*T* (b).

Figure 5 shows the molar mass distribution (e.g., *M*_*n*_, *M*_*w*_, *M*_*z*_, and *M*_*p*_) of the PLGA studied for the pellet and for the other times of degradation.

Based on the results presented, PLGA plates have a narrow (low polydispersity) and monomodal (only one peak) molar mass distribution. The pellet represents unprocessed PLGA, in which high molecular mass (e.g., 183407 ± 28895 Da) was identified. As shown in Figure 5, values of molar mass *M*_*n*_ decrease from pellet to 0 days of degradation, which can be explained by material processing during injection molding.

PLGA plates' degradation can be observed by mass loss during the time of contact with the phosphate buffer solution; however, the degradation speed between PLGA_low*T* and PLGA_high*T* was different. This behavior probably occurred due to the reduction of the amorphous phase of the polymers, since the molecular interaction of the solution with the copolymer is more propitious in the amorphous phase. According to Middleton and Tipton (2000) [12], in the first stage of degradation, there is diffusion of water in the amorphous regions of the polymer and hydrolytic cleavage of the ester bonds of the polymer chains. After much of the amorphous phase undergoing degradation begins the second stage in the crystalline phase; therefore, there is a percentage increase in the degree of crystallinity.

### 3.5. Scanning Electron Microscopy

**Figure 6:**
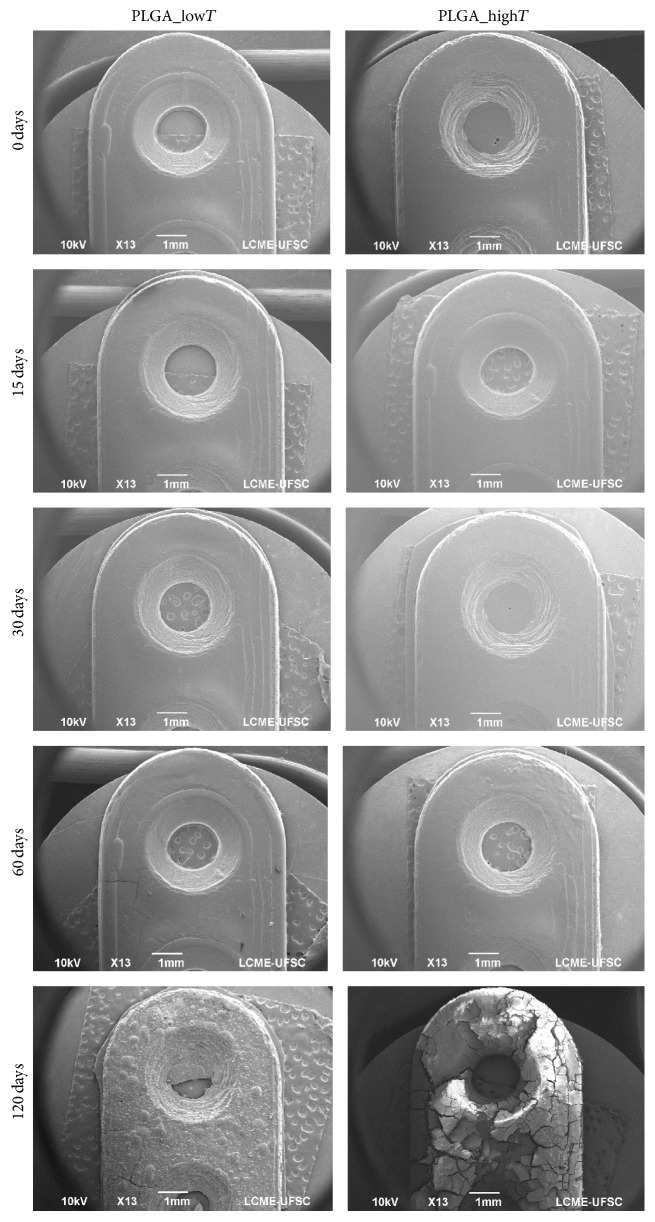
SEM images of the PLGA_low*T* and PLGA_high*T* plates at different degradation time (0, 15, 30, 60, and 120 days) (bar scale = 1 mm).

PLGA plates' degradation during the immersion period in phosphate buffer solution (PBS, 37°C) is qualitatively confirmed by the SEM images (magnification ×13), as shown in Figure 6.

**Figure 7:**
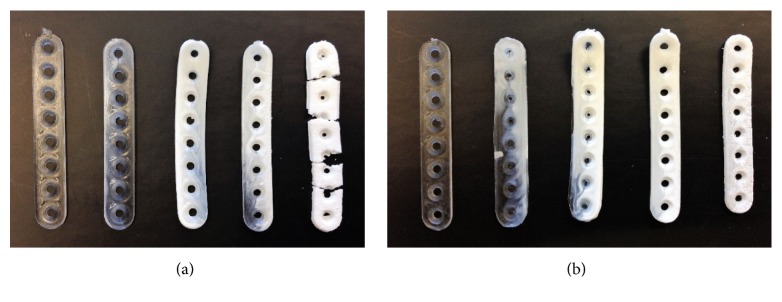
Visual appearance images of the degradation of the PLGA_low*T* (a) and PLGA_high*T* (b) plates at the different degradation points: 0, 15, 30, 60, and 120 days from left to right.

Figure 7 shows the material degradation process for PLGA_low*T* and PLGA_high*T*, which coincides with the results obtained from the SEM analyses.

Initially transparent PLGA plates become opaque when degradation process begins. The whitish aspect of the device is clear in the first 15 days in phosphate buffer solution, which is an indication of the influence of the degradation process, as a function of the organization of the chains during the degradation. During the process, the deformation and brittle feature of the material can be noted. After 120 days, it was possible to notice the crumbling of the material and the absence of mechanical properties. The whitish form can be noted in point 1 for PLGA_high*T*, which is less evident in PLGA_low*T*. That could indicate that PLGA_high*T* is more sensitive to hydrolytic degradation in less time.

## 4. Discussion

PLGA copolymer is a promising material for medical devices applications as craniofacial plates. Medical devices manufactured from aliphatic polyesters degrade through hydrolysis of the polymer backbone primarily through a bulk degradation process that includes decline of molecular weight, reduction in mechanical properties, and loss of mass. Hydrolytic degradation can be evaluated through molar mass (GPC), presence of polar groups or oligomers and monomers (FTIR), and changes in mechanical properties (DMA), transition temperature changes (DSC), and surface and geometry (SEM) [21–23]. This study reported the* in vitro* degradation of PLGA craniofacial plates tested in two different melt temperatures of the injection molding process (i.e., 240 and 280°C), corresponding to the processable limits of PLGA, at different time points. Physicochemical properties such as molecular weight and mechanical properties were monitored by FTIR, DSC, and DMA analysis. The results suggested that the property changes differ according to the injection molding temperature.

The flexural strengths for the PLGA plates studied in this work ranged from 1.9 ± 0.1 to 2.2 ± 0.1 GPa, which compared with the stiffness (*E*) of bone (*E*_bone_ ~ 6–20 GPa), metal (*E*_metal_ = 100–200 GPa), and poly(lactic acid) (11–72 MPa) [24], indicating a possible use of these plates under investigation in non-load-bearing body sites, such as for craniofacial bone fractures. However, bioresorbable plates PGA-based copolymers have higher degradation rate than other bioresorbable polymers, which limits the useful time of the devices [25, 26]. Both PLGA plates conditions (i.e., PLGA_low*T* and PLGA_high*T*) showed rapid degradation, regardless of the different characteristics of the microstructure during the degradation.

The evaluated mechanical properties of PLGA_low*T* and PLGA_high*T* showed suitable values at the beginning of degradation [27–30]. However, PLGA_high*T* plates reached flexural strength peak and maximum flexural strain after 15 days, as observed in Figure 1. A possible reason for this result is related to the diffusion effect order from PBS solution to PLGA plates: first, the diffusion occurs to lower molecular weight chains of PLGA and then to higher molecular chains. In addition, degradation of resorbable polymers begins from higher molecular weight to lower molecular weight chains over time on PBS solution. In addition, smaller polymer chains can rearrange and relax over time before larger chains degrade. Thus, the diffusion to the solution of lower molecular weight chains of PLGA prior to degradation of the higher molecular weight fractions renders the material stiffer after 15 days but with continuing decrease after degradation of the material.

Moreover, Table 3 shows crystallinity and thermal properties on degradation of the plates which could be associated with chains hydrolysis, diffusion, and erosion. At the beginning of the degradation, the phenomenon of surface erosion can be observed, where molar weight loss is exclusively from the outside to the inside of the material, where diffusion of the water molecules, for example, is slower than the release of fragments from the surface. In another case, the volumetric degradation occurs when water penetrates the polymer matrix homogeneously, causing hydrolysis throughout. In this event, there is a relationship between hydrolysis of the chains and their diffusion and erosion. If any disturbance occurs, this equilibrium may be undone and a variation of the mechanism known as autocatalysis via carboxylic and hydroxyl groups may occur. This autocatalysis in volumetric degradation causes an acid gradient in the inner part of the body, causing accelerated degradation to occur at this site compared to the surface. The oligomers generated in the central regions can easily diffuse to the surface. This effect, accompanied by the presence of acid products, may result in inflammatory reactions in* in vivo* cases. It is worth mentioning that the degradation of devices implanted in the human body, an object of interest in this work, tends to present an increase in the rate of diffusion and consequent degradation due to the body temperature around 37°C, variations in pH, and eventual efforts which may increase the probability of breaking connections [13, 18, 19, 31]. An increase in molecular weight will result in more covalent bonds and thus an increased number of entanglements and thereby increasing resorption/degradation time [32].

**Figure 8:**
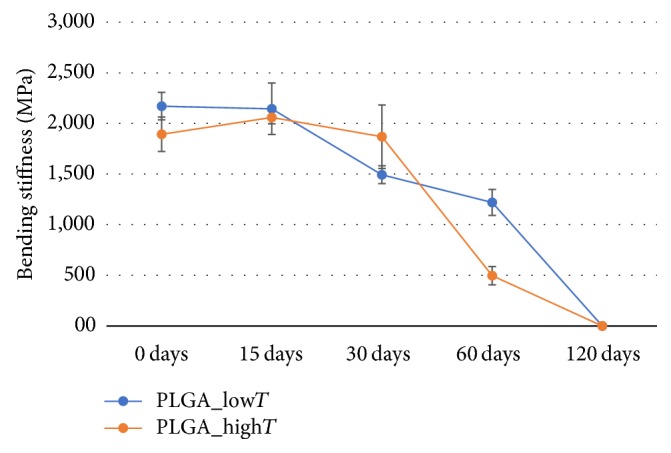
Bending stiffness along the hydrolytic degradation of PLGA_low*T* and PLGA_high*T* plates.

The decrease of properties (Figure 8) was evident at each time point of the degradation for PLGA_low*T*; however, viscoelasticity properties possibly influenced the dispersion for PLGA_high*T* in the first 60 days of degradation, which showed stable values of the properties found, with drop in the last two points.

In DSC curves, it was evident that the PLGA_high*T* plates would have greater propensity to crystallize than PLGA_low*T* plates due to their greater steric regularity along the polymer chain during processability [33]. Injection molded PLGA_high*T* and rapid cooling had the effect to reduce *T*_*g*_ by about 4°C. Furthermore, the cooling time (i.e., 90 s) of injection molding was the same for both plates' conditions. It means that the cooling rate to reach the temperature of the mold (23°C) was faster for PLGA_high*T* plates. Because of this difference, different crystalline phases were formed, which may have resulted in the formation of different sizes of spherulites and irregular crystals in polymer structure [26, 34, 35].

Moreover, several polymers properties that are important in terms of their processability and applications are directly related to the specific molar mass. That could be related to the fact that mechanical, chemical, and physical properties are drastically affected by the crystallinity and especially by the low and high molar mass fractions. Devices for this application need to be deeper investigated to overcome complications on manufacturing and designs that could influence the degradation rate after placement such as properties stability.

## 5. Conclusion

We have proposed a work limit of temperatures (low and high) on medical devices as PLGA craniofacial plates manufactured by injection molding and tested biomechanical function degradation of the two conditions of melt process temperature. Both working temperatures allowed producing craniofacial plates devices. At low and high temperature conditions (i.e., 240 and 280°C, resp.), the PLGA plates were evaluated for mechanical properties (apparent elastic modulus, maximum stress, and storage modulus) and crystallinity. The mechanical properties (i.e., 2.2 ± 0.1 and 1.9 ± 0.1 GPa of flexural stiffness) of the plate are suitable for osteosynthesis in non-load-bearing anatomical sites (e.g., craniofacial applications). The differences in crystallinity showed that we can choose the plate with degradation kinetics more suitable for the application. Based on all these results, we can conclude that the proposed process temperatures are adequate for the manufacture of PLGA craniofacial plates. In addition, the knowledge presented is useful to better understand the working limits of bioresorbable implants and the development of implant geometries with property control.

## Figures and Tables

**Table 1:** Injection molding parameters used to produce PLGA plates. All the production parameters were kept constant while varying the melt injection temperature (240°C for PLGA_low*T* and 280°C for PLGA_high*T*).

	PLGA_low*T*	PLGA_high*T*
Melt injection temperatures	240°C	280°C
Mold temperature	25°C	
Injection pressure	1500 MPa	
Holding pressure	25 MPa	
Injection time	2 s	
Cooling time	90 s	
Screw speed	100 rpm	

**Table 2:** Mechanical properties of injection molded PLGA 85/15 plates (mean ± standard deviation, *n* = 3): flexural stiffness, *E*; flexural strength, *σ*_*r*_; and maximum flexural strain, *ε*_*r*_  (^*∗*^*p* < 0.05).

		*E* (GPa)^*∗*^	*σ*_*r*_ (MPa)^*∗*^	*ε*_*r*_ (%)^*∗*^
PLGA_low*T*	0 days	2.2 ± 0.1	41.4 ± 11.8	2.6 ± 0.8
	15 days	2.1 ± 0.2	54.6 ± 3.9	3.5 ± 0.5
	30 days	1.5 ± 0.1	12.5 ± 7.7	1.3 ± 0.7
	60 days	1.2 ± 0.1	19.05 ± 0.8	2.8 ± 0.4

PLGA_high*T*	0 days	1.9 ± 0.1	30.1 ± 3.1	2.3 ± 0.2
	15 days	2.1 ± 0.07	42.3 ± 7.2	2.9 ± 0.6
	30 days	1.9 ± 0.3	24.5 ± 15.3	1.8 ± 0.7
	60 days	0.5 ± 0.1	4.1 ± 0.8	1.0 ± 0.1

**Table 3:** Degree of crystallinity (*X*_*c*_) and thermal properties measured by DSC: glass-transition temperature (*T*_*g*_), melting point (*T*_*m*_), enthalpy of cold crystallization (Δ*H*_*c*_), enthalpy of glass (Δ*H*_*g*_), and enthalpy of melting (Δ*H*_*m*_) (^*∗*^*p* < 0.05).

		*T*_*g*_ [°C]^*∗*^	*T*_*m*1_ [°C]	*T*_*m*2_ [°C]	*T*_*c*_ [°C]^*∗*^	Δ*H*_cc_ [J/g]^*∗*^	Δ*H*_*m*_ [J/g]^*∗*^	Δ*H*_*g*_ [J/g]^*∗*^	*X*_*c*_ [%]^*∗*^
	Pellet	60.4 ± 1.8	—	146.1 ± 0.1	—	—	33.4 ± 2.4	1.7 ± 0.4	35.7

PLGA_low*T*	0 days	56.0 ± 0.5	153.1 ± 0.3	156.6 ± 0.2	130.7 ± 2.4	−2.3 ± 0.6	4.3 ± 1.5	5.1 ± 0.5	7.1
	15 days	53.0 ± 3.2	153.8 ± 2.2	157.5 ± 1.8	131.0 ± 0.1	−8.9 ± 2.2	5.0 ± 2.6	5.9 ± 1.3	14.8
	30 days	51.8 ± 1.2	153.6 ± 0.9	156.4 ± 1.0	99.9 ± 7.7	−17.5 ± 3.1	14.5 ± 6.9	9.6 ± 4.0	34.1
	60 days	48.3 ± 0.3	138.7 ± 4.2	147.3 ± 5.4	87.4 ± 4.1	−35.3 ± 7.8	28.6 ± 6.8	7.1 ± 12.0	68.2
	120 days	41.7 ± 6.6	120.4 ± 0.2	133.5 ± 1.0	95.5 ± 0.1	−30.2 ± 0.9	19.7 ± 8.7	14.1 ± 3.8	53.3

PLGA_high*T*	0 days	52.7 ± 0.9	151.0 ± 2.2	157.9 ± 1.0	124.5 ± 4.2	−24.0 ± 8.1	17.9 ± 5.1	3.1 ± 0.3	44.7
	15 days	52.5 ± 3.1	154.1 ± 0.1	157.2 ± 2.0	127.1 ± 14.5	−9.9 ± 4.3	7.9 ± 3.0	9.1 ± 0.4	19.0
	30 days	52.6 ± 0.8	152.0 ± 0.1	156.6 ± 0.4	102.1 ± 8.3	−68.6 ± 3.6	19.1 ± 7.1	10.5 ± 0.5	93.7
	60 days	52.0 ± 1.1	153.7 ± 0.7	157.7 ± 1.7	89.0 ± 3.9	−47.9 ± 2.2	7.9 ± 7.0	11.8 ± 6.5	59.6
	120 days	41.6 ± 0.8	144.4 ± 2.8	152.4 ± 3.2	96.6 ± 1.3	−28.9 ± 6.9	23.2 ± 4.1	0.4 ± 0.1	55.7

**Table 4:** Absorption bands identified in the FT-IR assay characteristic of the functional groups present in the PLGA copolymer.

Absorption bands (cm^−1^)	Groups
3000–2850	CH, CH_3_ e CH_2_
1760–1745	C=O
1600–1500	O-C=O (oligomer)
1450–1370	CH_3_ and CH_2_
1350–1150	CH_2_ and CH
1300–1150	C-O
800–750	CH
